# Techno-economics of integrating bioethanol production from spent sulfite liquor for reduction of greenhouse gas emissions from sulfite pulping mills

**DOI:** 10.1186/s13068-014-0169-8

**Published:** 2014-12-05

**Authors:** Abdul M Petersen, Kate Haigh, Johann F Görgens

**Affiliations:** Department of Process Engineering, University of Stellenbosch, Cnr Banghoek and Joubert Street, 7600 Stellenbosch, South Africa

## Abstract

**Background:**

Flow sheet options for integrating ethanol production from spent sulfite liquor (SSL) into the acid-based sulfite pulping process at the Sappi Saiccor mill (Umkomaas, South Africa) were investigated, including options for generation of thermal and electrical energy from onsite bio-wastes, such as bark. Processes were simulated with Aspen Plus® for mass- and energy-balances, followed by an estimation of the economic viability and environmental impacts. Various concentration levels of the total dissolved solids in magnesium oxide-based SSL, which currently fuels a recovery boiler, prior to fermentation was considered, together with return of the fermentation residues (distillation bottoms) to the recovery boiler after ethanol separation. The generation of renewable thermal and electrical energy from onsite bio-wastes were also included in the energy balance of the combined pulping-ethanol process, in order to partially replace coal consumption. The bio-energy supplementations included the combustion of bark for heat and electricity generation and the bio-digestion of the calcium oxide SSL to produce methane as additional energy source.

**Results:**

Ethanol production from SSL at the highest substrate concentration was the most economically feasible when coal was used for process energy. However this solution did not provide any savings in greenhouse gas (GHG) emissions for the concentration-fermentation-distillation process. Maximizing the use of renewable energy sources to partially replace coal consumption yielded a satisfactory economic performance, with a minimum ethanol selling price of 0.83 US$/l , and a drastic reduction in the overall greenhouse gas emissions for the entire facility.

**Conclusion:**

High substrate concentrations and conventional distillation should be used when considering integrating ethanol production at sulfite pulping mills. Bio-wastes generated onsite should be utilized at their maximum potential for energy generation in order to maximize the GHG emissions reduction.

**Electronic supplementary material:**

The online version of this article (doi:10.1186/s13068-014-0169-8) contains supplementary material, which is available to authorized users.

## Introduction

Ethanol production can be integrated with a paper and pulp facility, however, the energy balance needs to be optimized due to the energy-intensive nature of both processes [1-3]. Energy-efficient integration of bioethanol production into processes such as the magnesium oxide (MgO) acid sulfite pulping process provides opportunities for the co-production of bio-energy products with existing pulp products. In sulfite pulping processes, such as those used by the Sappi Saiccor mill in Umkomaas, South Africa, the energy requirements for the pulping process are provided by concentrating the spent sulfite liquor (SSL), which contains dissolved lignocellulosic components, such as lignin derivatives and hemicellulose sugars, and feeding the resulting syrup to a recovery boiler [2]. The energy generated from the combustion of SSL is often not sufficient to satisfy the energy demands of the mill itself, and an additional fuel source, such as bark, biomass residues or coal, is needed [2,4]. If the sugar components in SSL are converted to ethanol, the calorific input to the recovery boiler would reduce, increasing the reliance on a supplemental fuel source, which in the case of Saiccor is coal. This demand for supplemental energy will be compounded with the energy demands of an integrated ethanol facility.

The energy demands of existing ethanol production from molasses or syrup come primarily from the ethanol separation and purification section of the process [5]. The energy demand of such distillation processes is directly related to the concentration of ethanol in the fermentation beer that serves as distillation feedstock [1,6]. In turn, this concentration is dependent on the concentration of fermentable sugars in the feed substrate (the SSL), and the efficiency of sugar conversion to ethanol during fermentation [6]. The fermentation of SSL poses challenges in both aspects, as the sugar concentrations of SSL (between 2 and 3% (w/w)) are lower than required [6-8]. In addition, the presence of toxins, such as organic acids and furans, create an inhibitory environment for the yeasts, which reduces fermentative activity [6,7,9]. Sugars represent 25 to 30% of the total dissolved solids (TDS), which implies that SSL sugars cannot be concentrated to more than 50 to 60 g/l. At this point, the TDS of 30% will become inhibitory to fermentation due to osmotic stress and media viscosity [6,7,9], even for detoxified SSL. These limitations imply that the SSL fermentation products will typically contain 20 g/l of ethanol, which is significantly less than the minimum of 40 g/l required for energy- efficient distillation [10].

Nonetheless, the fermentation of SSL on a laboratory scale has shown considerable success, using both native xylose fermenting strains and genetically modified (GM) strains. Nigam [7] conducted fermentation experiments using *Pichia stipitis* on an SSL sample derived from hardwood via a calcium oxide (CaO) sulfite process, and was able to achieve an ethanol yield of 70% (of total sugars) at a dissolved solids content of 26%, resulting in a maximum ethanol concentration of 20.5 g/l. In order to achieve this performance the strain was adapted through prior growth in SSL that was treated with alkaline detoxification. Without prior adaption, a sugar-to-ethanol conversion of 10% was achieved. A recent attempt using mutants of *P. stipitis* for fermenting hardwood SSL was made by Bajwa *et al*. [11]. While all sugars were used, the results show that an excess amount of ethanol was metabolized, since the ethanol yields only ranged from 60 to 65%. Using a GM strain (*Saccharomyces cerevisiae* 256ST), Helle *et al*. [9] was able to obtain yields of 85% and 76% on MgO-based SSL samples derived from eucalyptus wood, having dissolved solid contents of 20% and 30%, respectively. This was without detoxification, and final ethanol concentrations of 16.5 g/l and 22.5 g/l were attained.

The energy integration of ethanol production into a sulfite pulp mill is challenging because of the energy demands of the ethanol process and the reduction of the energy content of the SSL available as the boiler fuel. The reliance of the combined pulping-ethanol process on supplementary fuel sources would thus be increased. Coal is presently the default source of supplementary heat and power at Sappi Saiccor due to installed coal-fed boilers (CFB). However, the use of fossil fuels would devalue the net greenhouse gas reductions (NGHGRs) associated with the production of ethanol as a fossil fuel replacement. A study has shown that the NGHGR of United States-produced corn-ethanol, powered by fossil fuels, was 47%, compared to 74% for Brazilian-produced sugarcane-ethanol because the sugarcane residues were used to provide energy and export electricity [12,13]. Biomass-to-energy (BTE) systems for combined heat and power (CHP) are typically less efficient than coal-based systems due to the lower calorific value of biomass [14]. Consequently, the economic viability of energy production from biomass has only been realized when the cost for obtaining the biomass is low [15]. Large amounts of forestry residues are generated at pulp mills at approximately 460 kg (bark, branches, tops and leaves) per ton of wood chips processed [16]. The use of bark for energy generation is an established strategy for supplementing the energy needs of pulping mills in the United States [4]. Another option for renewable energy to supplement the energy demands of the pulp mill is the biodigestion of organic-rich effluents for the production of biogas [17].

The present study investigated strategies for integrating ethanol production into an MgO sulfite pulping plant, to achieve both the intended NGHGRs and economic viability, based on the flow rates of, and samples shipped from the Sappi Saiccor mill as industrial representatives. The Sappi Saiccor mill is an acid sulfite pulping mill situated in Kwazulu-Natal, South Africa, which produces virgin cellulose pulp from wood, primarily *Eucalyptus globulus.* Furthermore, various CHP scenarios were combined with the various ethanol production processes, to obtain an economically viable and environmentally beneficial scenario. The study aims to represent a conservative case regarding ethanol production, by considering flow sheet inputs from the SSL samples that were received with the lowest sugar concentration. In this way, the energy demands will be overestimated and the ethanol production will be underestimated.

Various flow sheets were considered, with the objective being to minimize the combined energy requirements of SSL concentration and ethanol distillation. All the ethanol production scenarios assume the use of an industrial yeast strain with the same (or superior) capabilities of the GM strain *S. cerevisiae* 256ST used by Helle *et al*. [9], and substrate detoxification as a conservative design step. The distillation of the crude products to anhydrous ethanol will also consider variants, such as multi-effect and conventional distillation (CON), which pose advantages in steam and electricity use, respectively [18].

## Results and discussion

### Chemical analysis

The chemical analysis for the sugars, organic acids, furans, total phenolic compounds and the total polymeric compounds (TPC) present in the SSL are shown in Table 1. The organic acids and furans are important indicators of the inhibitory nature of the SSLs as fermentation feedstock, and compared well to the literature [7]. The acetic acid was present at 4.26 (0.47%) to 9.02 g/l (0.99%), while previous reports indicated 9.3 g/l [7] and 0.8% [8]. The furfural concentration was measured at 0.239 g/l, compared to 0.2 g/l [7,8] that was previously measured. The concentration of the total phenolic compounds at 125.5 mg/l was lower than that reported by Marques *et al*. [8].

**Table 1:** **Results for chemical analysis of spent sulfite liquor samples (HPLC – high performance liquid chromatography; MgO – magnesium oxide; CaO – calcium oxide; SSL – spent sulphite liquor; HMF – hydroxyl-methyl-furfural; TPC – total polymeric compounds)**

	**Unit**	**MgO-SSL**		**CaO-SSL**	
**HPLC Measured**		**Amount**	**Error**	**Amount**	**Error**
Furfural	g/l	0.29	0.01	0.23	0.01
HMF	mg/l	4.50	0.50	6.00	2.00
Formic Acid	g/l	0.81	0.08	2.21	0.04
Acetic Acid	g/l	4.26	0.35	9.02	0.15
Glucose	g/l	1.38	0.06	2.78	0.06
Xylose	g/l	18.25	1.16	27.68	0.45
Arabinose	g/l	0.86	0.07	1.18	0.01
Phenolics	mg/l	48.55	2.86	23.85	1.40
Solids	%	10.95	0.05	14.39%	0.10%
Ash	% of Solids	10.34	1.25	8.76%	0.00%
Elemental Composition					
Carbon	%	5.78	0.25	8.41%	0.29
Hydrogen	%	10.32	0.36	9.46%	0.34
Nitrogen	%	0.59	0.16	0.23%	Maximum^a^
Sulphur	%	1.45	0.13	2.64%	Maximum^a^
Oxygen	%	81.86	Difference	81.18%	Difference
Calculated TPC	%	8.99%		13.70%	

^a^The elemental analyser measures organic sulphur and nitrogen by the SO_2_ and nitric oxides respectively, which are generated during combustion of the sample. The CaO ash generated during the combustion of Ca SSL absorbs acidic gasses [19], which then causes variability in the sulphur and nitrogen in this particular sample. Thus, the maximum value is reported as a conservative measure. Since the Ca SSL is only considered for biodigestion of the non-recalcitrant sugars, with no further processing, this result does not affect the outcomes of the simulations in any way.

The concentration of total sugars (glucose, xylose and arabinose) in the SSL was 20.49 g/l, which was lower than the measurements by Marques *et al*. [8] for the same stream from the Sappi Saiccor facility, and the previous measurement of 28.47 g/l from a previous shipment of samples (data not shown). Flow sheet analysis based on the measurements in Table 1 would thus represent a pessimistic estimation of the energy performance of an integrated ethanol facility, especially with regards to the concentration of ethanol in the fermentation beer.

### Evaluation of process energy considerations and greenhouse gas emission characteristics

The technical information describing the ethanol scenarios (refer to Table 2 for a description of the scenario names), with supplemental energy supply to satisfy the net demands of the integrated pulping-ethanol process, are presented in Table 3. Equations 1 and 2 were used to calculate the equivalent values of the coal required to generate the steam and electricity demands for the base case scenarios; at an excess of what is currently required by the pulping facility. The greater value between the equivalent amounts of coal determined for electricity and thermal energy defined the coal requirement for the respective scenario. This is because coal supplied at the lower equivalent value would result in a deficient generation of the utility that required the higher coal demand. Regarding the amounts of steam and electricity generated by the BTE system, these are in excess of the demands of the ethanol production scheme, and would partially fulfil demands of the pulping facility and displace the equivalent amount of coal. In these energy scenarios, if the amount of coal displaced is greater than the lower of the equivalents of coal determined, this would result in a deficit in the supply of that utility corresponding to the lower equivalent. In those cases (i.e. the BTE augmented schemes), the limiting displacement of coal was defined by the lower value of the equivalent’s values determined for steam and electricity.

**Table 2:** **Summarized description of scenarios considered (TDS – total dissolved solids; CON- conventional distillation; ME – multi-effect distillation; BG – biodigestion; BTE- biomass to energy)**

		**Ethanol Process Alternative**			
		**20% TDS, Convention Distillation**	**20% TDS, Multi-effect Distillation**	**30% TDS, Convention Distillation**	**30% TDS, Multi-effect Distillation**
**Energy Generation Alternatives**	**Coal Fired**	20-CON	20-ME	30-CON	30-ME
	**Coal Fired with Biodigestion**	20-CON-BG	20-ME-BG	30-CON-BG	30-ME-BG
	**BTE**	20-CON-BTE	20-ME-BTE	30-CON-BTE	30-ME-BTE
	**BTE with Biodigestion**	20-CON-BTE-BG	20-ME-BTE-BG	30-CON-BTE-BG	30-ME**-**BTE-BG

**Table 3:** **Analysis of effect of integration of ethanol production on energy characteristics (CON- conventional distillation; ME – multi-effect distillation; BTE- biomass to energy)**

	**Ethanol Cogeneration**				**Current Recovery**
**Scenarios**	**20%-ME**	**20%-CON**	**30%-ME**	**30%-CON**	
Concentrated SSL Flow rate (tons/hr)	135.21		95.94		
**Net Outputs**					
Bioethanol Production (l/hr)	2,555.63	2,556.35	2,321.48	2,321.48	
**Steam Utilities**					
Gross Steam Generation (tons/hr)	124.35	125.11	124.23	124.50	135.54
Total Steam Demand (tons/hr)	52.77	73.77	53.68	63.70	45.17
Total Additional Steam Utility Required (tons/hr)	18.79	39.03	19.82	29.56	
**Electricity Generation and Requirements**					
Gross Electricity Generation (MW)	13.39	13.46	13.37	13.40	14.60
Power and/or Ethanol Utilities (MW)	5.27	2.80	4.13	2.19	0.41
Total Additional Electricity Utility Required	6.08	3.53	4.95	2.99	
**Base Case - Coal Driven**					
Coal for Steam Demand (tons/hr)	2.59	5.38	2.73	4.08	
Coal for Electricity Demand (tons/hr)	8.08	4.71	6.59	3.99	
Required Supply (tons/hr)	8.08	5.38	6.59	4.08	
Net Greenhouse Gas Reduction Potential (tons/hr)	−23,159.77	−15,129.65	−18,300.52	−10,821.18	
**BTE Augmented Scenarios**					
Steam Supply (tons/hr)	75.80	75.80	75.80	75.80	
Electricity Generation (MW)	10.57	10.57	10.57	10.57	
Displacement of Coal (tons/hr)	−5.97	−5.07	−7.46	−6.38	
Utility Causing Maximum Coal Displacement	Electricity	Steam	Electricity	Steam	
Net Greenhouse Gas Reduction Potential (tons/hr)	18,720.18	16,034.64	23,579.43	20,343.11	
**Biodigestion - Coal Driven Scenarios**					
Required Supply (tons/hr)	7.32	4.32	5.63	3.25	
Net Greenhouse Gas Reduction Potential (tons/hr)	−20,904.11	−11,951.92	−15,449.50	−8,350.75	
**Biodigestion - BTE Augmented Scenarios**					
Displacement of Coal (tons/hr)	−6.73	−6.14	−8.42	−7.44	
Net Greenhouse Gas Reduction Potential (tons/hr)	20,975.84	19,212.37	26,430.45	23,523.50	

The energy demands of the ethanol production processes were compared in terms of distillation (multi-effect under vacuum versus conventional) and sugar concentration in the SSL feed. The heat integration potential of multi-effect distillation (ME) resulted in a reduction in the steam demand of 42.41%, compared to the CON scenarios. However, the ME scenarios also required 70.10% more electricity than the conventional scenarios, due to the demands of the vacuum pump between the beer and rectifier columns. Overall, ME needed 55.78% more coal equivalents than the conventional scenarios, primarily due to the high thermal energy demands of steam-based electricity generation. Higher sugar concentrations in the SSL with 30% TDS reduced the electricity and steam demands by an average of 16.93% and 9.40%, respectively, due to lower volumes of fluid processed per unit of ethanol produced.

The results in Table 3 shows that the extent to which excess energy supply from the BTE augmented system could substitute the existing coal consumption was limited by the electrical demand, in case of ME, and steam demand for the CON scenarios. Displacement of existing coal utilization with surplus BTE energy supply was 17.40% higher for the multi-effect scenarios than for the CON scenarios. For the base cases however, where all of the energy requirements were met by coal combustion, the CON scenarios were advantageous over the ME, due to lower coal requirements. The difference is due to the boiler pressure used in each energy generation scheme, which impacts the electrical generation efficiency. The pressure ratio of the CFB and BTE steam cycles were 6.1 and 10.6, respectively, which resulted in a specific electricity generation of 0.092 MW and 0.125 MW per ton of steam generated, respectively. Thus, per ton of steam produced, the BTE system generated more electricity than the CFB system, thus favoring scenarios with higher electrical demands.

The NGHGR of the base case scenarios (driven by coal only) were all negative, which implied that integration of ethanol production into the pulp mill would worsen GHG emissions over the process life cycle, compared to the pulping-only processes.,. As the global warming potentials ((GWPs) the − NGHGR) are in accordance with the coal required for the various scenarios, the lowest GWP of 10,821.18 kg CO_2(eq)_ was achieved by the 30-CON, while the highest GWP of 23,159.77 ton/hr CO_2(eq)_ was attained by the 20% solids-multi-effect scenario (20-ME). While additional energy supplementation through biodigestion reduced the coal consumption for the base scenarios by 0.90 tons/hr (14.95%), the reduction in the GWP potentials were not enough affect a positive NGHGR. The NGHGR values of the BTE augmented schemes are all positive, indicating that the integrated ethanol-pulping facility significantly reduced GHG emissions of the process life cycle, compared to the pulping-only facility. As these ratios are in accordance with the coal displacement potentials, the best NGHGR of 23,579.43 tons/hr CO_2(eq)_ was attained by the 30-ME-BTE scenario. When digestion is considered in conjunction with a BTE system, the coal displacements are typically improved by 0.96 tons/hr while the NGHGRs were improved by 14.90% on average.

### Economic results

**Figure 1:**
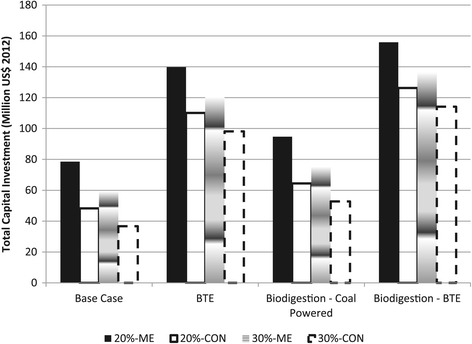
**Total capital investment.** (CON- conventional distillation; ME – multi-effect distillation; BTE- biomass to energy).

The total capital investment (TCI) of all scenarios considered is shown in Figure 1. The coal-only scenarios represented the lowest TCIs of the ethanol production processes, as the existing coal boiler will be used. The lowest capital investment of US$ 36,710,000 was for the 30-CON. This is due to the high sugar concentrations in the SSL feedstock resulting in a high ethanol concentration and thus lower volumetric throughputs and smaller equipment sizes. Furthermore, the equipment costs associated with the vacuum pump for ME is negated. Comparing the two distillation configurations shows that ME increased the TCI by 61.90%. Accordingly, the highest capital requirement was needed by the 20-ME scenario.

**Figure 2:**
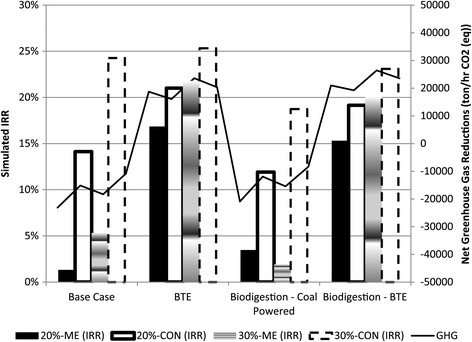
**Simulated IRR of the various scenarios in relation to net GHG reductions.** (CON- conventional distillation; ME – multi-effect distillation; BTE- biomass to energy).

Comparing economic viability, as measured by the internal rate of return (IRR) (Figure 2), it is shown to be strongly related to capital investment, as the IRR of the 30-CON (base) was in just short of 25%, while that of the 20% solids-multi-effect scenario (20-ME) (base) was just about 1%. The economic viability is also related to coal consumption, demonstrated by the increase in the IRR for scenarios with decreased coal consumption. Furthermore, it is shown that the higher yield of ethanol possible with a more diluted SSL substrate (i.e. dissolved solids at 20%) did not justify the additional capital and process costs compared to fermentation with 30% SSL.

The installation of a BTE system is shown to bear substantial capital costs, as shown by an increase of almost three-fold in the base capital costs of the 30-CON scenario when a BTE was installed. However, installation of a BTE for coal replacement generally increased the IRR of the combined ethanol-energy facility, thus justifying the additional capital costs. The BTE installation improved the economic viability of the 20-ME scenario the most, due to a lower increase capital requirement and substantial coal displacement, in contrast to intensive use of coal in the coal-only scenario.

The installation of biodigestion generally had a negative effect on the economic viability (in contrast to the installation of BTE) as shown by a decrease in the IRR. The IRR of the 20-ME scenario, however, increased by 2.17% due to biodigestion installation, because there was a minor effect on the overall capital requirements. The most severe was a decrease of 5.54% in the IRR for the 30-CON, due to the significant effect of capital requirements for biodigestion in this particular scenario. The combination of biodigestion with BTE augmented scenarios resulted in an average decrease of 1.83% in the IRR, compared to BTE augmented scenarios without biodigestion. Thus, inclusion of biodigestion is shown to have a negative effect on economic viability, since the savings it generates by coal supply reductions are not enough to justify the capital expenditure.

In Figure 2, the relationship between the IRR and the NGHGR is shown for all the process scenarios considered. For the coal-fed scenarios, the minor improvement in life-cycle reduction of GHG emissions that biodigestion offered was not economically justifiable. In addition, the significant improvements in GHG reductions that the BTE augmentation afforded generally caused an improvement in the IRR as well, which implies that the overall costs of a BTE system are cheaper than coal. Of the BTE scenarios, the 30-ME-BTE scenario had the highest net GHG emission reductions, while the 30-CON scenario had the best economic viability, due to its lower TCI. This pattern was also observed when biodigestion was considered with BTE.

### Analysis on plant scale and minimum ethanol selling price

A subsequent analysis was carried out on the effect of economies of ethanol production scale on the economic viability of the 30-CON-BTE-BG (BG-biodigestion) scenario. Again, citing the Sappi Saiccor facility as an example, the SSL throughput was doubled by assuming that both of the MgO SSL streams would be directed to ethanol fermentation, instead of fermenting one of the streams. The 30-CON scenario with digestion was selected for this analysis because it offered the greatest potential for environmental benefits, though it was not attractive for private investment (IRR less than 25%). The impact of the increase of scale by a factor of two on the minimum ethanol selling price (MESP), coal displacement, NGHGRs and IRR was determined.

**Figure 3:**
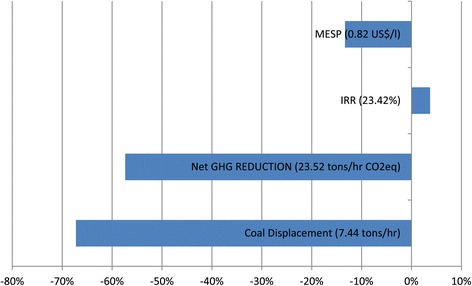
**Effect of plant scale on key variables.** Value in parenthesis is obtained for the small-scale process, while the bars show the percentage of change that the scale-up caused.

Doubling the rate of ethanol production resulted in an increase in electrical and thermal energy demands, which was met by the combination of biodigestion and BTE (Figure 3). The energy scheme was also capable of providing a surplus of energy to displace coal at a rate of 2.12 tons/hr, although it was less than coal displacement at the smaller scale. Doubling the ethanol production scale also reduced the NGHGR by 49.98% when compared to the smaller scale. The reduction in the NGHGR and coal displacement was because the scale of the BTE system remained static, while the demands of energetic utilities had increased significantly due to the increase in scale by a factor of two. Thus, the resulting IRR only improved by an absolute amount of 3.73%, (16.13% relative) ,which is a mild effect when compared to previous reports on the effect of economies of scale [20], where a scale-up of about two-fold in production capacity in an integrated ethanol plant resulted in about an 80% increase in economic viability.

The MESP required to produce an IRR of at least 25%, with an 80% probability of surpassing this benchmark, was determined for the smaller and larger production scale of the 30-CON scenario. The MESP of 0.82 US$/l for the smaller scale was unfavorable, relative to Brazil (0.67 US$/l) and the United States (0.61 US$/l), although it improved by 9.6% to 0.71 US$/l for the larger scale. In a South African context, the MESP of the small-scale production compares favorably with the preliminary price of 1.07 US$/l for first-generation ethanol determined in the South African Department of Energy biofuel strategy [21].

### Sensitivity of the net greenhouse gas reductions potential, internal rate of return and the minimum ethanol selling price to initial substrate concentrations

**Figure 4:**
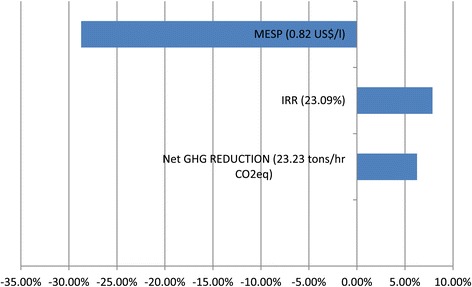
**Effect of initial SSL sugar concentration on key variables.** Value in parenthesis is resulted for the diluted feed, while the bars shows the percentage of change that the concentrated substrate caused.

Comparison of SSL-fermentation scenarios was based on a measured total sugar concentration of 20.49 g/l in SSL before concentration (Table 1), which was shown to be lower than the sugar concentration measured in samples of previous shipments. The impact of higher sugar concentrations in the SSL on the economic viability and environmental benefits (NGHGR) of the 30-CON-BTE-BG scenario was thus determined. The impact of the increase in sugar concentration of the ethanol production rate was determined for the IRR, MESP and NGHGR (Figure 4).

The increase of 39% in the sugars concentration in SSL significantly improved economic viability, as reflected in an increase of 7.86% in the IRR and reduction of the MESP by 27.83% - to a value of 0.58 US$/l. Operating the digesters in the sulfite pulping process at the highest solids (woodchips) loading would thus increase the sugar concentration in the SSL and subsequently benefit the associated ethanol process. At an expected average sugar concentration of 24.61 g/l in the SSL, an IRR of 27.03% can be achieved if the ethanol selling price based on average international prices of ethanol is assumed. Regarding the NGHGR, a marginal increase of 6.25% can be expected, due to the increased production of ethanol and the subsequent increase in displacement of gasoline.

## Conclusion

The preferred scenario for integration of ethanol production from MgO-SSL into a sulfite mill was SSL with 30% TDS from the existing multi-effect concentrator as feedstock, and purifying the ethanol from the resulting fermentation broth using CON. The capital costs of this process was lowest when using coal to supply all of the energy demands of the integrated pulping-ethanol process, although there was an increase in greenhouse gas emissions. Maximum use of thermal and electrical energy generation from bark (combustion) and evaporator effluents (biodigestion) to replace coal consumption provided satisfactory economic viability and significant greenhouse gas reductions. In addition, the chemical oxygen demand (COD) of the effluent discharge was reduced. The effect of scaling the ethanol production by considering two MgO-SSL streams as fermentation substrates, instead of just one, had improved the IRR, but also reduced the NGHGR.

Through the rigorous methodology of flow sheet development, a scheme for integrating the production of ethanol, as well as thermal and electrical energy from biowastes, into a pulping mill was developed. Economically, the optimized flow sheet is highly competitive against first-generation ethanol in South Africa, even at a small production scale, and could compete with international ethanol prices at larger scales. Less conservative estimations than applied in the present investigation may further improve the IRR, or lower the MESP, of the resulting process.

In this study, it was assumed that the effects of the inorganic chemicals that are used to condition the SSL for fermentation on the recovery boiler would be significant and thus the costs associated with these effects were conservatively estimated. Thus, it is recommended that a detailed study be carried out to determine these effects, so that an energy efficient and cost effective means of mitigating the effects can be found if those effects are shown to be significant enough to intensify the operation of the recovery boiler system. These studies however, are likely to be carried out privately, as the knowledge of the exact operation of these systems is not in the open literature.

## Methodology

### Overview of the acid sulfite process

**Figure 5:**
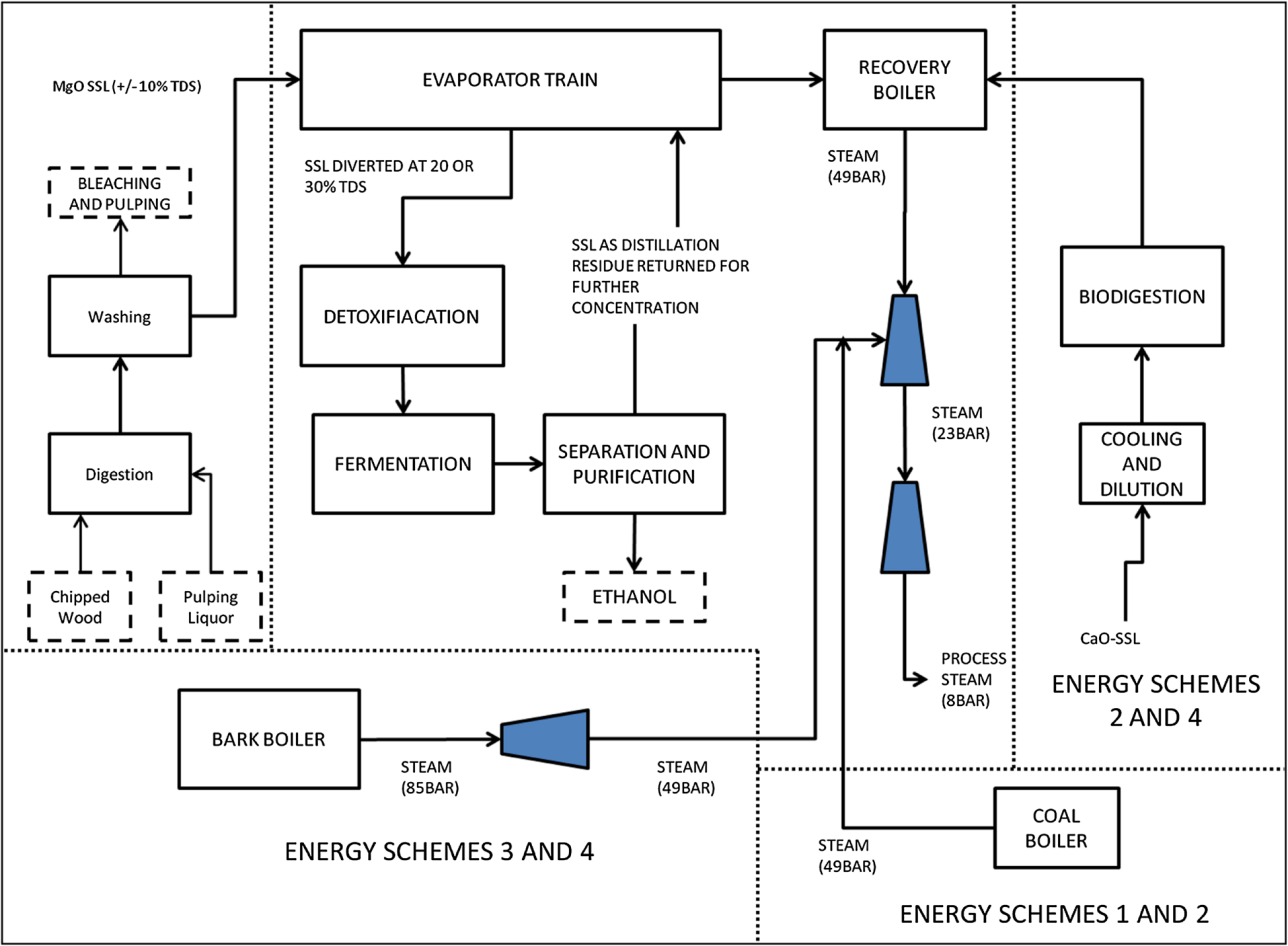
**Schematic overview of the scenarios for ethanol integration and energy generation schemes.** (TDS-total dissolved solids; MgO – magnesium oxide; CaO – calcium oxide; SSL – spent sulphite liquor)

The pulping process begins by de-barking, then chipping wood-logs. The woodchips are mixed with water and pulping liquor, followed by pulping with SO_2_ injection in the digesters, to solubilize lignin and hemicellulose sugars [22]. The crude pulp discharged from the digesters is washed with water in a counter-current vacuum filter system and the clean pulp is sent for chemical bleaching and mechanical pulping, while the wash-filtrate, referred to as SSL, is processed by concentration and combustion [22]. A brief schematic flow is provided in Figure 5.

The Sappi Saiccor facility has three acid sulfite lines, two of which are MgO-based (MgO1 and MgO2), and one that is CaO-based [22]. The SSL from the two MgO lines are concentrated in separate trains of evaporators to form boiler feed syrup for energy production and pulping chemicals recovery. Of the SSL from the CaO line, 70% is sold to an adjacent lignosulfonates processing facility, while the rest is discharged as an industrial effluent (personal communication with B. Chizwanza and C. Reddy). Thus, the MgO-SSL line is more appropriate as a substrate for ethanol production, while the effluent portion of the CaO-SSL is available as substrate for biodigestion. In the present study only one of the MgO-SSL streams is considered for ethanol production, while the processing of the second MgO-SSL line remains unaltered.

### Materials and analysis

Samples of the SSLs were shipped from the Sappi Saiccor facility and analyzed for monomeric sugars, organic acids, furans [7,8] and phenolic compounds [23] using high performance liquid chromatography (HPLC analysis), as previously used for acid hydrolysates [24,25]. In order to determine the TDS, the samples were dried at 110°C over a sufficient period for the samples to be bone dry. The dried samples were then combusted at 725°C in order to determine the ash content.

The elemental composition of the SSL was also analyzed using a LECO TrueSpec Micro elemental analyzer (LECO® Corporation, Worldwide) to determine the carbon, hydrogen, nitrogen and sulphur contents, with the oxygen content determined by difference. As the organic content of SSL typically comprises polymeric sugar/lignin compounds, extractives, monomeric sugars, organic acids and furans [8], the chemical formula and quantity of the TPC were determined as the difference between the total atomic make-up (shown by the elemental analysis) and the total portion of these elements found in the measured compounds.

### Process development

Given the low concentration of sugars generally found in SSL, the liquor requires concentration prior to fermentation. In cited literature [6,7,9], the range of TDS concentration generally considered for fermentation is 20 to 30%, where the sugar concentrations would typically be in the range of 50 to 70 g/l. Thus, the ethanol plant would be integrated into the existing evaporation trains at a sulphite mill, by diverting the SSL syrup for ethanol feedstock from after the concentrating stage, where the TDS concentration is 20% or 30%. The process flow sheet for the various integration strategies is shown in Figure 5.

The first process step after concentration in the ethanol production process is detoxification. Detoxification with lime is deemed impractical on an industrial scale, due to the large quantities of gypsum created [6]. Instead, detoxification with soluble compounds, such as sodium hydroxide or ammonium hydroxide, are better alternatives to avoid these issues and both have previously been shown to be effective at improving fermentability [26]. The choice depends mainly on the effect of these chemicals on the ANDRITZ chemical recovery system (recovery boiler) (ANDRITZ AG, Graz, Austria) that is presently operating at the Sappi Saiccor facility [27]. Detoxification with sodium hydroxide and sulfuric acid was selected, as the resulting sodium sulphate in the SSL residues after fermentation will have a minor impact on the existing system, since the ANDRITZ recovery scrubber (ANDRITZ AG, Graz, Austria) already has sections dealing with water-soluble ashes [27]. However, as a conservative measure to account for the associated costs, it will be assumed that the scrubber will be modified with a bag filter to capture the extra load of condensing alkali salts in order to avoid corrosion. The capital and maintenance costs of the bag filters will be taken from the National Renewable Energy Laboratory (NREL) [28,29] reports.

The detoxified SSL will be filtered to remove flocculants that might have formed during detoxification, before splitting about 3 to 4% of it to acclimatize the yeast to toxic environments during cell production. The fermentation performance of a robust recombinant strain of *S. cerevisiae*, reported to convert more than 80% of the pentose sugars in an untreated SSL sample to ethanol [9], was assumed for concentrated detoxified SSL. Specifically, conversions of 85% and 76% of the sugars were considered for the 20% and 30% substrates respectively [9], at cell concentrations of 3 to 5 g/l. Thus, the inclusion of detoxification is a conservative measure to assure high yields, as it had previously been shown that conversions without detoxification could be reduced by 60% [7]. With regards to the electrical energy needed to agitate the fermentation tanks, half of the specific rate of 11 MJ_e_ per ton of substrate that estimated from the NREL reports [28] for hydrolysis and fermentation was considered, as there were no hydrolysis reactors in this study. Heat removal is done with chilled water at 15-20°C provided by a chiller, which operates at a coefficient of performance of seven [30].

The beer from fermentation is first heated and flashed at 0.86 bar to remove the CO_2_, which is sent to a knockout drum to recover ethanol, followed by ethanol recovery through scrubbing of the vapor from the knockout drum [31]. The liquid effluents from the flash vessel, knockout drum and scrubber are combined and further combined with the fermentation beer into the distillation train [31]. This train can be either conventional, where the beer stripper and rectifier columns are both atmospheric, or a multi-effect system where the beer stripper column is under vacuum, to allow the heat released by the rectifier condenser to effect the reboiler of the beer stripper [32,33]. In either case, the distillation system produces vapor phlegm that is 91% ethanol, which may be dehydrated with molecular sieves to an anhydrous quality of 99.7% ethanol [31], depending on market demand.

The syrup produced as the beer stripper bottoms is returned to the evaporation train for concentration of the remaining TDS content to 60%, and subsequent combination with the MgO SSL syrup not used for fermentation, as feed to the recovery boiler. The recovery boiler produces superheated steam at 49 bar pressure, which is sequentially expanded to 23 bar and 8 bar, to provide the required live steam in the plant. Heat recovery from the stack gasses from the boilers is currently sufficient to reduce the temperature of the stack gasses to 135°C. As further information about the energy recovery scheme was not available, it is assumed that the recommended energy recovery measures, such as an economizer after the boiler and an air pre-heater, are applied as components in recovering heat from the stack [2,4]. Further measures for recovery boiler operation, like minimizing the excess air fed to meet the environmental regulatory requirement of the oxygen content being 6% in the stack gas, are also assumed to apply [34].

### Establishment of the reference case for the current utility usage

Estimations of industrial flow-rates of SSL citing the Sappi Saiccor facility are conservatively estimated to be 285 ton/hour for each of the three process lines (for MgO1, MgO2 or CaO). Only one of the MgO lines are considered for ethanol production, again giving a conservative economic estimate in terms of the economies of scale of ethanol production, and minimizing the impact of ethanol production on the overall pulping-ethanol energy balance. The reference case on which to base ethanol production scenarios is the current processing of SSL, which is concentration in a multi-effect system followed by energy and chemicals recovery in the recovery boiler. The data from Perin-Levasseur *et al*. [35] was used to simulate the six-effect evaporator for SSL concentration in Aspen Plus [36], (Aspen Technology, Inc, Massachusetts, United States of America (USA)) together with simulation of the recovery boiler. From these simulations, the following reference points for the existing MgO-SSL evaporator train and recovery boiler could be established: 1) the concentration of TDS of the streams leaving each evaporator, so that concentrated SSL streams with 20% TDS or 30% TDS could be withdrawn and compared in ethanol production scenarios; 2) the heat duty of each evaporator; 3) the amount of steam produced by the recovery boiler that is currently consumed by the mill and 4) the amount of electricity generated by the turbines, and that is consumed by equipment like the boiler feed pump and combustion air fans. The net available electricity from power generation is presently consumed by the mill.

### Simulation of ethanol scenarios

The four ethanol scenarios were simulated as the combination of two possible TDS concentrations in the SSL feed from the existing evaporator train, and two possible distillation schemes. For each scenario, the syrup is returned from the distillation section to the remaining stages of the evaporation trains, and then subsequently fed to the recovery boiler system. The necessary simulation of the pumping costs will all be accounted for in the simulation. Thus, from the simulation of ethanol scenarios in Aspen Plus [36] and the subsequent comparison with the values determined for the reference case, the sets of information attained and calculated are as follows: 1) the production rate of ethanol, chemical requirements and energy generation of the recovery boiler system from the syrup returned after distillation; 2) the minimum hot and cold utility duties calculated by recording the heating and cooling demands from the simulations and processing these through Pinch Point Analysis using the ICheme spreadsheet [37]; 3) the net steam and electrical utility requirements calculated as the difference in the increased utility demands of the integrated plant to the reference case, and the deficit in the utilities generated by the recovery boiler of the integrated facility, compared to the recovery boiler of the reference case.

### Simulation of external energy generation schemes

The combined heat and power plants that provide energy to both the pulping and ethanol processes, using either biomass or coal, were simulated in Aspen Plus [36], according to the approach recommended by the Aspen Plus manual [38]. The chemical characteristics of two fuel types are presented in Table 4. The thermal energy recovery scheme from combustion (described in the process development section) for the recovery boiler is assumed to extend to the existing CFB boilers and the new BTE augmentation system proposed for supplementation of the process energy supply [39]. Figure 5 depicts how the flow of the energy streams from these systems is integrated into the overall systems of the various energy schemes.

**Table 4:** **Chemical characteristics of bark and coal**

**Proximate Analysis %**		
**Material**	**Coal [** 40 **]**	**Bark [** 41 **]**
Moisture	5.00	47.50
Fixed Carbon	55.40	6.60
Volatile Matter	27.00	41.50
Ash	12.60	4.40
**Elemental Analysis (% Moisture Free)**		
Hydrogen	4.42	5.52
Carbon	70.63	49.12
Nitrogen	1.79	0.38
Sulphur	0.72	0.04
Oxygen	9.18	36.56
Ash	13.26	8.38

The thermal and electrical utilities of the combined pulping-ethanol plant, in excess of what would be provided by the existing recovery boilers, was quantified in terms of the equivalent amount of coal required for the CFB boilers. Thus, the Aspen Plus model for coal-to-energy was simulated with a range of coal flow rates, using the sensitivity analysis feature. From the data generated, a set of linear equations that related the coal-fed versus steam and net electricity generation were produced, which was then used to determine the equivalent amount of coal needed for the utilities. Thus, the steam generated is determined by Equation 1 and net electricity generated is determined by Equation 2:

1 $$ \mathrm{STEAM} = 7.249 \times \mathrm{COAL} + 0.008 $$

2 $$ \mathrm{ELECTRICITY} = 0.755 \times \mathrm{COAL}\ \hbox{-}\ 0.021 $$

For the BTE system, a flow rate of 34 tons/hour of bark was considered, based on current supply from the digestion capacity of 8,000 tons of wood a day [22], and the rate of bark generation at 150 kg per ton of wood prepared [16]. In order to achieve a high electrical generation efficiency in the BTE system, this boiler will operate at a pressure of 85 bar [42]. This steam will be expanded in a back-pressure turbine to 49 bar so that it can be injected into the current steam expansion-extraction system.

Supplemental energy for the recovery boiler was also obtained by biodigestion of a portion of the CaO-SSL, presently disposed as effluent. The CaO-SSL was diluted with mild process wastewaters, to reduce the COD from a value of >170 to 79 g/l so that the stream would be suitable for biodigestion microbes [17]. The CaO-SSL was also cooled by means of a heat exchanger to a temperature of 40°C to ensure a temperature of about 35°C after dilution, which is optimal for microbe activity [17]. The microbes generate biogas by fermenting the non-recalcitrant components in an up-flow anaerobic sludge blanket (UASB) reactor which, for a typical SSL stream, reduces the COD by 20% [17]. The biogas is then used to supplement the fuel of the recovery boiler, since the recovery boilers at Sappi Saiccor have ports through which supplemental gas is fed.

### Scenario development

The ethanol production scenarios could consider two substrate concentration levels (20% or 30%), which would either be combined with ME or CON distillation, and thus four ethanol production schemes (20-ME, 20-CON, 30-ME, 30-CON) are possible. The energy supply scheme for all the ethanol scenarios could either consider additional coal (base case), the BTE augmentation system, supplementation of biogas generated from CaO-SSL biodigestion or a combination of BTE augmentation and supplementation of biogas. The coal-driven scenarios are described in their base form (for example, 30-CON), while the biodigestion- and BTE-driven scenarios have the description amended with BTE or BG (for example, 30-CON-BTE, or 30-CON-BG). With all renewable energy supplementation included, the descriptions are amended with BTE-BG (for example, 30-CON-BTE-BG).

### Technical evaluation

The various ethanol scenarios were evaluated based on the amount of additional coal needed to drive the process. If a biomass boiler was considered, then the steam or electricity generated in excess of the demands of the ethanol integration was converted to an equivalent amount of coal that would be replaced. The effect of supplementing the recovery boiler with biogas generated from the biodigestion of the CaO-SSLon the overall energy balance, in terms of the amount of coal it replaces was also evaluated.

### Net greenhouse gas reductions

Since this study involves renewable energy products from processes driven with fossil and renewable energy sources, the determination of the NGHGR is imperative. The NGHGR has been defined as the negative the GWP, that is, NGHGR = −GWP. Life-cycle assessment techniques adapted for process evaluations were applied to compare the GWP of each scenario [43-45]. The system boundary for the GWP determination included all of the affected sub-systems in the processes, which was begun with the diversion of the MgO-SSL from the evaporation train to ethanol production. Thus, it included the chemical manufacture and transport, coal mining and transport, ethanol production, utility generation and coal displacement. As this study is concerned with comparing various process technological routes in an integrated facility rather than end products, the basis for comparison will use the reference flow method [43], instead of functional units.

Inflow-outflow data for the Life Cycle Inventory was recorded from the Aspen Plus simulations, while the data for the specific GWP associated for each input and output was extracted from the Sima-Pro [46] database generated by the CML 2001 method [47]. Data for greenhouse gas emissions for the coal supply included the emissions from coal mining in South Africa, which was taken from Zhou *et al*. [48], and the emissions from the transport of coal were extracted from the GREET (The Greenhouse Gases, Regulated Emissions, and Energy Use in Transportation) [49] database (Argonne National Laboratory, Chicago, USA). The ethanol production was assumed to replace an equivalent amount of gasoline [50], and the emissions resulting from that amount of gasoline (specific CO_2(eq)_ taken from GREET [49]) was subtracted from the system emissions, as per the substitute method [43,50]. The method described in the Ecoinvent database manuals [51] was then used to calculate the overall emissions for the systems of each scenario. Regarding the interpretation, a positive NGHGR means that the integration of ethanol production reduces greenhouse gas emissions for the combined pulping-ethanol plant, while a negative value indicates that the integration of ethanol actually increases global warming.

### Economic evaluation

The process models were used to construct financial models in order to assess the economic viability of the various technical scenarios considered. These financial models were based on Monte Carlo economics (MCE), so that the indicator of economic viability, such as the IRR or the MESP, is reported as an aggregated value that accounts for the effects of fluctuations on economic viability [52,53]. Thus, if the IRR is reported, which is primarily for comparing scenarios, it is an average value resulting from the numerous iterations involved in MCE. When the MESP is reported for the most technically and economically desirable scenario, it is reported as the selling price needed to produce an IRR of 25%, with a probability of 80% or more of achieving this IRR. An IRR of 25% represents the return level for private investment [54], while a failure rate of a probability of 20% is generally considered to be the maximum threshold for a target investor [55]. Detailed descriptions of the procedures that were followed for the capital estimation, establishment of the base financial model and implementation of MCE, and estimating economic parameters, are given in the support information file, as Additional file 1. The Simetar Excel Add-in [56] software (Simetar^©^, Texas, USA) was used for the Monte Carlo risk analysis.

The data used to populate the economic model is shown in Table 5 for the static and stochastic variables. Regarding fuel prices, it is not evident from the South African Biofuel Pricing Strategy (SABPS) [21] whether the pricing strategies developed for biofuels include second-generation ethanol from existing industrial process streams. Thus, an average first-generation ethanol price was calculated from United States- and Brazilian-based ethanol fuel prices, with no added premiums. In the section of the study where an MESP is calculated for the most desirable scenario, the future fluctuations of the basic fuel price (BFP) were simulated to base the forecasts of the future MESPs, because the SABPS stipulates that the first-generation biofuel price is related to the BFP. Thus, given that the future SABPS might include second-generation biofuels, the MESP calculated here is assumed to follow the projections of the BFP.

**Table 5:** **Data for inputs to MCE (US – United States; TDS – total dissolved solids; BFP – Basic Fuel Price; PPI – Producers Purchases Index)**

**Data for Static Value Inputs**						
**Item**		**Cost Basis**	**Value**			
General Running Costs and Miscellaneous Expenses [52]		US$/litre	0.032			
Added Maintenance Costs of Recovery Boiler [28]		US$/litre	0.003			
Total Chemical Cost for 20% TDS		US$ (per annum)	2,850,292			
Total Chemical Cost for 30% TDS		US$ (per annum)	3,808,123			
Delivery Price of Coal to Sappi Saiccor (Personal Communication)		US$/ton	115.8			
**Data for Stochastic Value Inputs**						
	**Electricity [** 57 **]**	**Brazilian Ethanol [** 57 **]**	**US Ethanol [** 57 **]**	**BFP [** 58 **]**	**PPI [** 57 **]**	**Interest Rate [** 57 **]**
**Year**	**US$/kWhr**	**US$/l**	**US$/l**	**US$/l**		**%**
2003	0.032	0.252	0.337	0.245	124.8	15.16
2004	0.039	0.254	0.422	0.337	127.7	11.31
2005	0.044	0.375	0.463	0.535	132.4	10.64
2006	0.048	0.508	0.674	0.676	142.6	11.14
2007	0.04	0.467	0.524	0.604	158.2	13.08
2008	0.04	0.52	0.587	0.838	180.8	15.12
2009	0.045	0.45	0.449	0.474	180.7	11.8
2010	0.054	0.612	0.483	0.601	191.6	9.91
2011	0.087	0.867	0.683	0.836	207.6	9
2012	0.106	0.666	0.611	0.865	220.5	8.78

## Additional file

Additional file 1:
**Economics Support Information File.** This file is a Microsoft Word File detailing the methodology followed for establishing the economic model and super imposing a Financial Risk Assessment on the model.

## Abbreviations

BFP: Basic Fuel Price
BTE: Biomass-to-Energy by Combustion
CaO: Calcium Oxide
CON: Conventional Distillation
GM: Genetically Modified
GWP: Global Warming Potential
IRR: Internal Rate of Return
ME: Multi-Effect Distillation
MESP: Minimum Ethanol Selling Price
MgO: Magnesium Oxide
NGHGR: Net Greenhouse Gas Reduction
SSL: Spent Sulfite Liquor
TCI: Total Capital Investment
TDS: Total Dissolved Solids
TPC: Total Polymeric Compounds
