# Supplementary material for: Techno-economics of integrating bioethanol production from spent sulfite liquor for reduction of greenhouse gas emissions from sulfite pulping mills
Source: Biotechnol Biofuels. 2014 Dec 5;7:169. doi: 10.1186/s13068-014-0169-8 (PMC4267141; doi:10.1186/s13068-014-0169-8)
Supplement: Additional file 1: — Economics Support Information File. This file is a Microsoft Word File detailing the methodology followed for establishing the economic model and super imposing a Financial Risk Assessment on the model. [file 13068_2014_169_MOESM1_ESM.docx]

**Support information file for: *Techno-economics and Greenhouse Gas Reduction of Integrating Bio-Ethanol Production from Spent Sulphite Liquor and Energy Generated from Bio-wastes at Sulphite Pulping Mills***

Author: Abdul M Petersen

Supervisor: Johann F Görgens

Affiliation:

Department of Process Engineering, University of Stellenbosch, Stellenbosch, South Africa

****This support Information file will form part of the appendix for the PhD Thesis of the author****

# Economic Assumptions

The parameters under which the economic evaluations of the scenarios were carried out are as follows:

- To simplify the economic analysis, the equity is assumed
- The life of the plant is 25 years and the period of analysis is 20 years, with 8000 operating hours per annum. The plant will have a salvage value of 20%, and the depreciation will be determined linearly from the initial value of the plant to the salvage value.
- The South African Company Tax rate of 28% applies.
- The working capital is 5%

# Capital Cost Estimation

## Equipment Costs

1. The capital cost of major equipment was estimated from literature using Equation 1. The values of the parameters in Equation 1 are Table 1.

C_ME_ = RC*(SP_S_/SP_R_)^SF^*IF*(CEPCI_FY_/CEPCI_RY_)

Where C_ME_ – Capital Estimation of Major Equipment

RC – Reference quoted price

SP – Scaling Parameter, simulated (S) and reference value (R).

SF – Scaling Factor

IF- Installation Factor

CEPCI – Chemical Engineering Plant Cost Index, of the first year of analysis (FY), and reference year (RY).

1. Aspen Icarus[1] was used to estimate the costs of generic equipment (C_GE_) such as pumps, turbines, compressors, flash tanks and process heaters and coolers.
2. The Total Equipment Costs (TEC)

TEC = ∑C_ME_ + ∑C_GE_

1. The Balance of Plant (BOP) [2], which estimates the costs of piping, instrumentation and wiring, is then calculated as:

BOP (%) = 0.8867 / (Biomass Higher Heating Calorific Input (MW))^0.2096^

1. The Total Installed Costs (TIC) are calculated as:

TIC = TEC+BOP+SD+W

Where: SD – Site Development, 13.5% of TEC [3]

W – Warehouses, 1.5% of TEC [3]

1. Total Fixed Costs (TFC) is calculated as

TFC = PC + FE +OC + C + O + TIC

Where: PC – Prorateable Costs

FE – Field Expenses, 10% of TFC [3]

OC – Office and Construction, 20% of TFC [3]

C – Contingency 10% of TFC [3]

O – Other Costs 10% of TFC [3]

1. Finally, the Total Investment Capital (TC) is calculated as

TC = TFC+WC

Where, WC – Working Capital, 5% of TFC. [3]

1. A location factor of 0.90 relative to a USA value of 1 is assumed on the Total Investment Capital [4].

Table 1: Parameters for Equipment Capital Estimate

| **Unit** | **Scale Parameter** | **Base Value USD** | **Base Year** | **Base Price** | **Base CEPCI** | **Scale Factor** | **Installation** | **Source** |
| --- | --- | --- | --- | --- | --- | --- | --- | --- |
| Detoxification Reactor | kg/hr Hydrolysate | 268762 | 2000 | 100144 | 392 | 0.71 | 1.40 | [5] |
| Neutralisation Reactor | kg/hr Hydrolysate | 268762 | 2000 | 100144 | 392 | 0.71 | 1.40 | [5] |
| Filter Press | t/h solids | 21 | 2000 | 1 285 736 | 392 | 0.60 | 2.40 | [5] |
| Seed Fermenters’ Coil | Heat Duty | 245 | 1997 | 4658 | 387 | 0.83 | 1.20 | [5] |
| Seed Fermenters | Volume m3 | 727 | 2000 | 149345 | 392 | 0.51 | 1.20 | [6] |
| Seed Holding Tank | Volume m3 | 872 | 2000 | 175626 | 392 | 0.51 | 1.20 | [6] |
| Fermentation Cooler | Heat Duty | 2800 | 1997 | 3054 | 387 | 0.78 | 2.10 | [5] |
| Fermentation Tank | Volume m3 | 3596 | 2000 | 539848 | 392 | 0.51 | 1.20 | [6] |
| Water scrubber | kg/h total feed | 25325 | 2000 | 127848 | 392 | 0.78 | 2.75 | [6] |
| Distillation columns | t/h ethanol | 29 | 2010 | 3327914 | 560 | 0.60 | 2.40 | [3] |
| Molecular sieve | t/h ethanol | 22 | 2010 | 2920000 | 560 | 0.60 | 1.80 | [3] |
| Boiler | t/h steam | 100 | 2010 | 31250000 | 560 | 0.73 | 1.00 | * |
| Heat Exchangers | Area m2 | 167 | 2010 | 44200 | 560 | 0.68 | 2.86 | [7] |
| Digester | m3 | 1 | 2010 | 714 | 560 | 0.91 | 1.00 | [8] |
| Chiller | Heat Duty kW | 1 | 2002 | 299 | 396 | 0.80 | 1.00 | [9] |
| Bag-house | Kmol Flue gas | 12935 | 2000 | 1784255 | 392 | 0.58 | 1.50 | [5] |

## Preparation of stochastic Simulation

The method described here is a summary of the method found in Richardson et al[10], and Amigun et al[11].

1. The raw data for stochastic variables (i) is used to derived time dependant linear equations, or an average valued indices, where from future values can be projected from

TDTV_i (t=2003...2012))_=m_i_∙t+c_i_

or

EV_i (t=2003,2012)_=Average_i_ (t=2003-2012)

Where: TDTV - time dependant trend value

m&c - trend line gradient and intercept.

EV - expected value

1. The residuals associated with each historical data point for each variable (Res_i_) will be calculated as the difference between the measured variable and its associated trend/expected value

Thus: Res_i, (t=2003...2012)_=MHV_i(t)_ – TDTV_i(t)_

OR

Res_i, (t=2003...2012)_=MHV_i(t)_ – EV_i(t)_

Where: MHV - measured historic value

1. The relative variances (V) associated with each residual and the trended value will be calculated as:

V_i (t=2003....2012)_=Res_i(t)_/TDTV_i(t)_

OR

V_i (t=2003....2012)_=Res_i(t)_/EV_i(t)_

1. The multivariate empirical distribution (MVEMP) characterising the measured variances will be used to simulate a vector of simulated variances (SV).

Thus: {SV_i_} _t=2012...2031_=MVEMP(V_i, t=2003...2012_)

1. Future yearly values will be simulated either as future time dependant trend value (FTDTV) or future expected values (FEV) using the formulas determined for the trend lines in Step (1)

FTDTV_i ,t=2012...2031_=TDTV_,i ,t=2012...2031_

OR

FEV_i, t=2012...2031_=EV_i, t=2003...2012_

1. The simulated future variance will then be combined with the future yearly value to calculated the stochastic Forecasted Economic Input (SFI).

SFI_i, ,t=2012...2031_= FTDTV_i, t=2012...2031_ + FTDTV_i, t=2012...2031_*SV_i, t=2012...2031_

OR

SFI_i, ,t=2012...2031_= FEV_i, t=2012...2031_ + FEV_i ,t=2012...2031_*SV_i, t=2012...2031_

1. Simulation of the Operating time (T) would be accomplished with the GRKS distribution with a 10 day variation around the average operating time of 8000 hours.

Thus T=GRKS(7760, 8000, 8240)

## Summary of Financial model for Calculating the Key Economic Variables

1. Simulating the Operating Expenses (OE) or Operating Incomes (OI)

If stochastic (i): OI_i, t_ or OE_i, t_ = Flow rate_i_*T*SFI_i, t_

If not stochastic (j) : OE_j, t_ = Base value_j_ *SFI_PPI, t_/PPI_t=2012_

Base values are given in Table 3 in the paper. They would be scaled with the quantity they are quoted in relation with.

1. Interest Calculations

Accrued Interest_t_ = (Negative cash balance)_t-1_*SFI_interest_

Interest Earned_t_ = (Positive cash balance)_t-1_*Interest on Positive Bank Balance (Table 3)

1. Net Profit/loss_t_ = ∑OI_i, t_ + Interest Earned_t_ - ∑OE_i, t_ – Accrued Interest_t_

NOTE: In South Africa, tax is paid on interest earned at these scales. Please see the SARS website for further information.

1. Net Cash Income/Deficit = Net Profit/loss_t_ – Depreciation_t_.
2. Dividends_t_ = Net Cash Income_t_ * 40%
3. Tax_t_=Net Cash Income_t_*28%
4. Cash Flow_t_ = Net Cash Income_t_ - Tax_t_ - Dividends_t_ OR = Net Cash Deficit
5. Cash Balance_t_ = Cash Balance_t-1_ + Cash Flow_t_
6. Assets_t_ = Plant Value_t-1_-Depreciation_t_+Land Value + Cash Balance_t_ (IF >0)
7. Liabilities = IF Cash Balance_t_ <0, Cash Balance_t,_ else = 0
8. Owners Equity_t_ = Assets_t_ – Liabilities_t_
9. Delta Net Worth_t_ = Owners Equity_t_ - Owners Equity_t-1_
10. Present Value_t_ = (Delta Net Worth_t_ + Dividends_t_)/(1+Discount Rate_(=12.64%)_)^t^
11. Net Present Value = -Total Capital Investment (TIC)+∑Present Values
12. For IRR – Solve for the discount Rate to yield a zero Net Present Value

# References

1. Aspen Technology Inc: **Aspen Plus®, Aspen Icarus®**. 2008.

2. Kreutz TG, Larson ED, Liu G, Williams RH: **Fischer-Tropsch Fuels from Coal and Biomass**. *25th Annual International Pittsburgh Coal Conference* 2008(August).

3. Humbird D, Davis R, Tao L, Kinchin C, Hsu D, Aden A, Schoen P, Lukas J, Olthof B, Worley M, D. Sexton and DD: **Process Design and Economics for Biochemical Conversion of Lignocellulosic Biomass to Ethanol Process Design and Economics for Biochemical Conversion of Lignocellulosic Biomass to Ethanol**. *National Renewable Energy Laboratory* 2011(May).

4. Perry RH, Green DW: *Perry’s Chemical Engineers' Handbook McGraw-Hill*. 7th Editio. Handbook McGraw-Hill; 1997.

5. Aden A, Ruth M, Ibsen K, Jechura J, Neeves K, Sheehan J, Wallace B, Montague L, Slayton A, Lukas J: **Lignocellulosic Biomass to Ethanol Process Design and Economics Utilizing Co-Current Dilute Acid Prehydrolysis and Enzymatic Hydrolysis for Corn Stover Lignocellulosic Biomass to Ethanol Process Design and Economics Utilizing Co-Current Dilute Acid Prehyd**. *National Renewable Energy Laboratory* 2002(June).

6. Leibbrandt NH: **Techno-Economics Study for Sugarcane Bagasse to Liquid Biofuels in South Africa: A Comparison between Biological and Thermochemical Process Routes**. *PhD Dissertation Department of Process Engineering University of Stellenbosch, University of Stellenbosch* 2010.

7. Al-Riyami BA, Klimes J, Perry S: **Heat Integration reftrofit analysis of a heat exchanger network of a fluid catalytic cracking plant**. *Applied Thermal Engineering* 2001, **21**:1449-1487.

8. Zamalloa C, Vulsteke E, Albrecht J, Verstraete W: **Bioresource Technology The techno-economic potential of renewable energy through the anaerobic digestion of microalgae**. *Bioresource Technology* 2011, **102**:1149-1158.

9. He B, Setterwall F: **Technical grade paraffin waxes as phase change materials for cool thermal storage and cool storage systems capital cost estimation**. *Energy Conversion and Management* 2002, **43**:1709-1723.

10. Richardson JW, Lemmer WJ, Outlaw JL: **Bio-ethanol Production from Wheat in the Winter Rainfall Region of South Africa : A Quantitative Risk Analysis**. *International Food and Agribusiness Management Review* 2007, **10**:181-204.

11. Amigun B, Petrie D, Görgens J: **Economic risk assessment of advanced process technologies for bioethanol production in South Africa : Monte Carlo analysis**. *Renewable Energy* 2011, **36**:3178-3186.
